# Family Involvement Interventions on Fear of Cancer Recurrence Management Among Women With Breast Cancer and Their Caregivers: A Systematic Review and Meta‐Analysis

**DOI:** 10.1111/jocn.17790

**Published:** 2025-05-14

**Authors:** Xiaofan Bu, Ling Jiang, Doris Y. P. Leung

**Affiliations:** ^1^ School of Nursing The Hong Kong Polytechnic University Hung Hom, Kowloon Hong Kong China; ^2^ Department of Nursing The Affiliated Suzhou Hospital of Nanjing Medical University Suzhou China

**Keywords:** breast cancer, family involvement interventions, fear of cancer recurrence, meta‐analysis, systematic review

## Abstract

**Background:**

Family strengths can be used to help families adapt to hardship and strain. However, meta‐analytic evidence of the effectiveness of family involvement interventions on fear of cancer recurrence (FCR) in women with breast cancer and their caregivers is lacking.

**Objective:**

To evaluate the effectiveness of family involvement interventions on FCR in women with breast cancer and their caregivers and to identify the characteristics of effective family involvement interventions.

**Method:**

Ten electronic databases were searched from database inception to October 2023. The updated Cochrane risk‐of‐bias tool was used to assess the quality of the included randomised controlled trials (RCTs). Data analyses were executed with Revman 5.3 software, and subgroup analyses were performed on the basis of interventional dosage. The Preferred Reporting Items for Systematic Reviews and Meta‐Analysis 2020 checklist was employed to provide guidance.

**Results:**

Seven studies were included in the review, and six were included in the meta‐analysis. The main contents included content related to the disclosure of disease‐related feelings/worries/concerns/experiences, education/psychological support plus some disclosure and education/counselling based on disclosure content. The results of the meta‐analysis showed that family involvement interventions have large short‐term positive effects on relieving FCR in women with breast cancer. The pooled results of subgroup analysis demonstrated that compared with usual care, education/psychological support plus some disclosure is ineffective, and disclosure alone has a moderate‐to‐large effect size, whereas disclosure with education or counselling targeting the specific needs of participants has an extremely large effect size. Only one study focused on FCR in caregivers, with an unfavourable result.

**Conclusions:**

Family involvement interventions, especially those using disclosure combined with education or counselling targeting their specific needs, have considerable short‐term effects on women's FCR alleviation. However, the evidence in caregivers is insufficient. Only a few interventional studies targeting patients and caregivers exist. Further high‐quality RCTs with follow‐ups are encouraged.

**Patient and Public Contribution:**

No patient or public contribution.

**Summary**

- What does this paper contribute to the wider global clinical community?
  ○ The main contents of effective family involvement interventions included content related to the disclosure of disease‐related feelings/worries/concerns/experiences, education/psychological support plus some disclosure and education/counselling based on disclosure content.
  ○ Family involvement interventions, especially those using disclosure to express feelings, worries, concerns, or experiences, combined with education or counselling targeting the specific needs of participants, showed remarkable short‐term effects on FCR alleviation in women.
  ○ Further high‐quality family involvement RCTs for women with breast cancer and their caregivers with follow‐ups are encouraged to generate adequate evidence.

## 1 Introduction

Breast cancer has become the most frequently diagnosed cancer in women in the world. Approximately 2.3 million new breast cancer cases were reported in 2020 worldwide (Sung et al. 2021). Anyone diagnosed with cancer may experience cancer recurrence. People who are diagnosed with cancer always worry that their cancer will return or progress. Fear of cancer recurrence (FCR) may become a permanent fixture in these people (Soriano et al. 2019a, 2019b), especially those whose cancer has been treated successfully and has high survival rates. FCR is a top unmet need in women with breast cancer (Bu et al. 2022; Fan et al. 2023). It refers to ‘fear, worry, or concern relating to the possibility that cancer will come back or progress (Lebel et al. 2016). The key characteristics of clinical FCR are persistent high levels of preoccupation and hypervigilance to bodily symptoms lasting for at least three months (Mutsaers et al. 2020). FCR is usually triggered by treatment‐related factors, including follow‐up appointments and treatment side effects; psychological factors; and media reminders (Mutsaers et al. 2016; Niu et al. 2019; Wang et al. 2025). Once it is triggered and poorly addressed, it tends to remain stable across disease trajectories for a long time (Schapira et al. 2022). When the level of FCR is high or clinically significant, women with breast cancer and their caregivers experience intrusive thoughts about the potential opportunities for cancer progression for a long time or till the end of their life; such thoughts, in turn, may deteriorate their quality of life and increase the risk of psychological problems (Cohee et al. 2017; Tran et al. 2022). In patients with breast cancer, FCR has a median proportion of as high as 47.9% (Fan et al. 2023).

FCR is a prevalent unmet need in cancer caregivers as well (Smith et al. 2022; Webb et al. 2023). In caregivers of women with breast cancer, FCR has a prevalence of 19%–64.5% and a pooled prevalence of 45% (Bu et al. 2022; Bu et al. 2025; Janz et al. 2016; Perndorfer et al. 2019; Soriano et al. 2021; Wang et al. 2021). In caregivers, FCR was found to be related to poor adjustment, quality of life, and emotional/mental functioning and a negative meaning of illness (Smith et al. 2022). Women with breast cancer and their caregivers focus mostly on the possibility of cancer recurrence, and a moderate positive correlation between FCR in women with breast cancer and their partners has been frequently reported in the literature (Muldbücker et al. 2021; Emily C. Soriano et al. 2019a, 2019b). FCR in one family member has a reverberating effect on other family members. FCR in caregivers may exacerbate poor outcomes in patients with cancer, either by reinforcing patients' concerns about recurrence or by compromising caregivers' ability to support patients (Smith et al. 2022). Therefore, families need to be viewed as a system that may be in need of supportive care. Numerous studies have recommended considering patients and caregivers as a unit and providing them with support (Janz et al. 2016; Soriano et al. 2021). The family is a social system and is considered the main social nucleus of individuals (McCubbin et al. 1980; Yoshimochi et al. 2018). It forms an integral part of the life and well‐being of patients who are at their most vulnerable when they are ill. Family strength is the ability to engage in family matters and exert coping capacity on the basis of a strong and resilient cohesion in family members; such cohesion can be used to help families adapt to hardship and strain (Lee and Han 2024).

Family‐based interventions are those that involve or collaborate with family members as part of various intervention components, and this involvement or collaboration affects intervention aims and outcomes (Ali et al. 2023). Although family involvement interventions have been tested on FCR management in women with breast cancer, their results were inconsistent, with some reporting significant positive results (Huang 2018; Zhang et al. 2019) but others reporting insignificant results (Northouse et al. 2005). Several reviews on FCR management are already available, but none analysed the effect of family involvement interventions in women with breast cancer and their caregivers. Previous reviews focusing on FCR management in women with breast cancer reported favourable results but usually involved patients and healthcare providers and not family members (Dawson et al. 2016; Lyu et al. 2022; Park and Lim 2022). Other reviews on family involvement interventions mainly focused on relationships; overall quality of life; physical health; psychological health in terms of anxiety, depression, well‐being, and body image; and social adjustment instead of FCR management (Li et al. 2023). The literature has found that interventions that promote family involvement can improve patient outcomes (Mackie et al. 2018). Meanwhile, given the high FCR levels and interplay of FCR in women with breast cancer and their caregivers, many studies have suggested considering women with breast cancer and their caregivers as pairs and intervening with them as an emotional and interdependent unit to provide cancer care (Boehmer et al. 2016; Janz et al. 2016; Soriano et al. 2021). Therefore, the present systematic review and meta‐analysis was conducted to (1) provide causal evidence with high quality from existing RCTs on family involvement interventions for women with breast cancer and/or their caregivers; (2) comprehensively estimate the effects of the RCTs on FCR in women with breast cancer and/or their caregivers; (3) compare the effects of different intervention contents; and (4) explore directions for future family involvement interventions.

## 2 Methods

### 2.1 Design

This systematic review was conducted and reported in accordance with the Preferred Reporting Items for Systematic Reviews and Meta‐Analysis (PRISMA) 2020 statement (Page et al. 2021). It was registered in the International Prospective Register of Systematic Reviews in 2023 (registration number: CRD42023480820). The PRISMA 2020 checklist was used to provide guidance for the reporting of the present systematic review (Supplementary File 1).

### 2.2 Search Strategies

Seven English language databases (PubMed, Embase, the Cochrane Central Register of Controlled Trials, Web of Science, PsycINFO, Ovid and CINAHL) and three Chinese language databases (CNKI, Wan Fang Data and Sinomed) were searched from inception to October 24, 2023. The search terms ‘caregivers’, ‘breast cancer’, ‘recurrence’, ‘fear’ and ‘trial’ were combined in each database by using free‐text terms and Medical Subject Headings where available. The detailed search strategies in Pubmed are shown in Table 1.

**TABLE 1:** Search strategy.

1	(‘breast’[MeSH Terms] OR ‘breast’[All Fields] OR ‘mammary’[All Fields]) AND (‘neoplasms’[MeSH Terms] OR ‘carcinoma’[MeSH Terms] OR ‘tumour*’[All Fields] OR ‘carcinoma*’[All Fields] OR ‘neoplasm*’[All Fields] OR ‘cancer*’[All Fields])
2	‘fear’[MeSH Terms] OR ‘Uncertainty’[MeSH Terms] OR ‘fear’[All Fields] OR ‘afraid’[All Fields] OR ‘worr*’[All Fields] OR ‘Uncertainty’[All Fields]
3	‘back’[MeSH Terms] OR ‘recurrence’[All Fields] OR ‘disease progression’[All Fields] OR ‘disease free survival’[All Fields] OR ‘back’[All Fields] OR ‘progress*’[All Fields] OR ‘recur*’[All Fields] OR ‘relaps’[All Fields] OR ‘reoccur*’[All Fields] OR ‘return*’[All Fields] OR ‘spread’[All Fields]
4	‘caregivers’[MeSH Terms] OR ‘spouses’[MeSH Terms] OR ‘carer*’[All Fields] OR ‘caregiv*’[All Fields] OR ‘care give*’[All Fields] OR ‘famil*’[All Fields] OR ‘parent*’[All Fields] OR ‘spouse*’[All Fields] OR ‘partner*’[All Fields] OR ‘husband*’[All Fields] OR ‘child*’[All Fields] OR ‘son’[All Fields] OR ‘daughter*’[All Fields]
5	‘RCT’[All Fields] OR ‘random*’[All Fields] OR ‘trial*’[All Fields] OR ‘group*’[All Fields] OR ‘intervention*’[All Fields] OR ‘experiment*’[All Fields] OR ‘control*’[All Fields]
6	#1 AND #2 AND #3 AND #4 AND #5

Abbreviation: MeSH, Medical Subject Headings.

### 2.3 Eligibility Criteria

Inclusion criteria were set by following PICOS domains. Studies meeting all the following criteria were included in this review:

1. Participants: Participants of the studies included women with breast cancer and their caregivers.
2. Intervention: Any type of intervention involving patients and caregivers.
3. Controls: Any type of control group.
4. Outcomes: FCR in either women or caregivers or in both as outcomes.
5. Study designs: Randomised controlled trials.

However, studies meeting any of the following criteria were excluded from this review:

1. Did not specify the type of cancer.
2. Included a mix of patients with cancer but lacked subgroup analysis for breast cancer.
3. Without available full‐text articles.

### 2.4 Literature Screening

Records from searches were imported into an EndNote library (EndNote X9.1), and duplicate studies were removed. The remaining records were transferred to an Excel spreadsheet (Microsoft, 2003). Screening was conducted by two independent reviewers who assessed the article titles, abstracts and full texts. Articles that did not meet the established inclusion criteria were excluded. Any disagreements between the two reviewers were resolved by discussion or in consultation with other investigators.

### 2.5 Data Extraction

Data extraction was completed by two reviewers (one reviewer extracted data from the included studies, and another cross‐checked the extracted data). The characteristics of each study (country and sample size), participants (caregivers' relationship with patients and patients' characteristics, such as type of cancer, age, stage and treatments and attrition rate), interventions (such as theoretical framework and detailed characteristics of the interventions in the experiment) and patients' and caregivers' outcomes and measurements were extracted.

### 2.6 Risk of Bias Assessment

Two reviewers independently assessed the methodological quality of all included trials. In case of doubt, the final decision was determined through discussion or consultation with other reviewers. The updated Cochrane risk‐of‐bias tool (ROB‐2) was used to assess the quality of the included RCTs (Higgins et al. 2019). The ROB‐2 tool includes five domains of bias: ‘risk of bias arising from the randomisation process’, ‘risk of bias due to deviations from the intended interventions’, ‘missing outcome data’, ‘risk of bias in the measurement of the outcome’ and ‘risk of bias in the selection of the reported result’. After assessing the risk of bias of each study, the studies were categorised into ‘low risk of bias’, ‘high risk of bias’ or ‘some concerns’. The overall risk of bias in each study was accordingly assessed as ‘high risk’, ‘low risk’ or ‘some concerns’.

### 2.7 Data Analysis

Review Manager Version 5.3 was employed to perform statistical analyses. The standardised mean difference (SMD) and SDs with 95% confidence intervals (CIs) were used to calculate continuous variables measured with different tools across studies. Initially, a fixed effects model was applied in the data analysis. Heterogeneity across studies was assessed by using *I* ^2^ statistics. For the chi‐squared test, *p* < 0.10 was considered statistically significant, indicating heterogeneity. *I* ^2^ values of 0%–40%, 30%–60%, 50%–90% and 75%–100% were regarded as not important, moderate heterogeneity, substantial heterogeneity and considerable heterogeneity, respectively (Higgins et al. 2019). If the *I* ^2^ value was greater than 50%, then the random‐effects model was chosen to summarise the evidence on the effects of family involvement intervention in the RCTs included in this study under the assumption that they have different true treatment effects regarding potentially existing heterogeneity between studies due to different types of interventions, frequencies, duration, numbers of sessions and measurement tools (Borenstein et al. 2010).

Subgroup analyses were conducted in accordance with the intervention characteristics. The *p* values for between‐group comparisons were calculated, and *p* < 0.05 was regarded as statistically significant. Sensitivity analysis was performed by excluding one study at a time to examine whether the results could have been influenced by a single study. The findings of studies that were not comparable and could not be included in the statistical pooling were presented narratively. Publication bias was not evaluated because only six studies were included in the meta‐analysis (Dalton et al. 2016).

## 3 Results

### 3.1 Search Results

**FIGURE 1:**
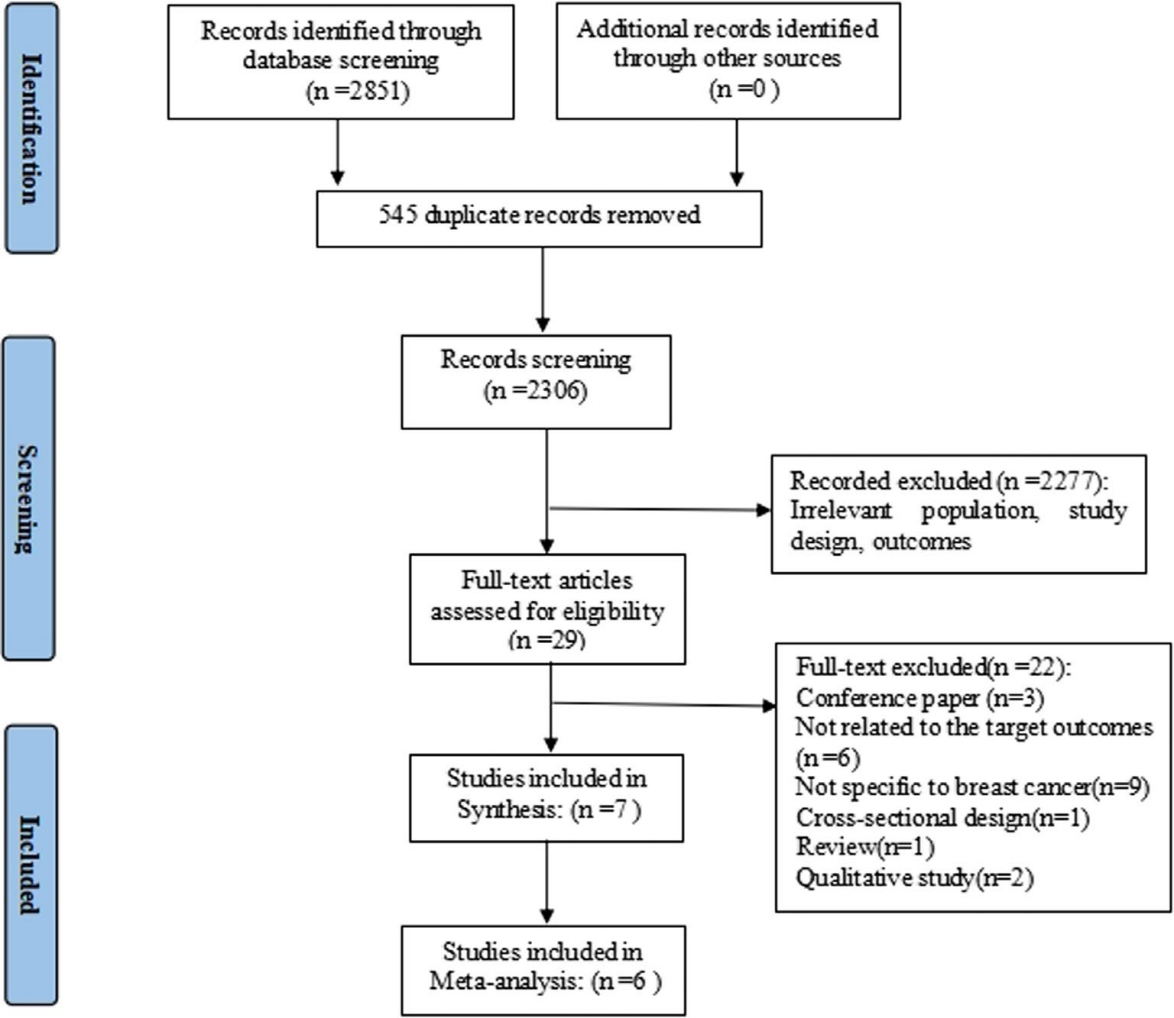
Flow diagram of study selection. [Colour figure can be viewed at wileyonlinelibrary.com]

A total of 2851 articles were identified. After 545 duplicates were excluded, the titles and abstracts of 2306 studies were reviewed, with 29 full‐text articles reviewed for eligibility. Seven studies met the eligibility criteria. A flow diagram of study selection is presented in Figure 1.

### 3.2 Study Characteristics and Participants

In the seven included studies, six were conducted in China and one in the USA (Table 2). The seven studies included 610 women–caregiver dyads. The sample size of the studies ranged from 24 to 182 dyads. Four studies (Huang 2018; Li and Zhu 2022; Northouse et al. 2005; Tan 2020) reported the cancer stage (I–IV) of the women patients. The majority of the women patients were undergoing chemotherapy. All six studies in China involved the spouses or partners of patients with breast cancer as the caregivers, whereas the one in the USA involved family members as the caregivers. The dropout rate ranged from 0% to 24.18%. The mean age of the female patients, reported in six studies (Chen et al. 2022; Huang 2018; Li and Zhu 2022; Northouse et al. 2005; Tan 2020; Zhang et al. 2019), ranged from their 30s to 50s, whereas that of the caregivers, reported in two studies (Huang 2018; Northouse et al. 2005), ranged from their 40s to 50s. Although family caregivers were involved in the interventions, only one study (Northouse et al. 2005) reported outcomes in women with breast cancer and their caregivers, whereas the remaining six studies reported outcomes in women with breast cancer only. All the included studies assessed FCR in patients, whereas only one study also assessed FCR in caregivers (Northouse et al. 2005). The details of the characteristics of the studies and included participants are shown in Table 2.

**TABLE 2:** Characteristics of the studies and participants in the seven included studies.

Author	Country	Research aim	Caregivers' characteristic			Patients' characteristic			
			Sample size of the dyads	Relationship	Age of caregivers (M ± SD)	Age of patients (M ± SD)	Stage of cancer	Treatments received by patients	Attrition (rate)
Northouse et al. 2005	USA	To evaluate the effectiveness of a family‐based intervention on quality of life and other psychosocial outcomes in patients with advanced breast cancer and their family caregivers	IG: 94, CG: 88	Family member	54.0 ± 11.0	52.0 ± 14.0	III, IV	Chemotherapy or a combination of therapies, hormonal therapy alone, or bone marrow transplant	IG: 21, CG: 23 (24.18%)
Huang 2018	China	To evaluate the effects of family synchronous cognitive therapy on recurrent fear, anxiety, depression and coping styles in patients with breast cancer	IG: 50 CG: 50	Spouses	IG: 45.72 ± 11.28, CG: 46.15 ± 10.78	IG:43.81 ± 8.17 CG: 44.45 ± 9.28	I–IV	Undergoing chemotherapy	IG: 2; CG: 3 (5%)
Zhang et al. 2019	China	To explore the effect of marital self‐disclosure intervention on FCR in young patients with breast cancer	IG: 31 CG: 31	Spouses	/	IG: 38.13 ± 4.39; CG: 37.87 ± 4.62	/	Undergoing chemotherapy	IG: 1 (1.61%)
Tan 2020	China	To explore the effect of spouse's support education combined with strengthened psychological support on psychological resilience and fear of disease progression in patients with breast cancer	IG: 25 CG: 25	Spouses	/	IG: 53.63 ± 2.15; CG: 53.89 ± 2. 11	I–III	Undergoing chemotherapy or radiotherapy	0
Chen et al. 2022	China	To explore the influence of self‐disclosure intervention on the mood of patients with breast cancer	IG: 45 CG: 45	Spouses	/	IG: 47.35 ± 8.39; CG: 48.69 ± 9.13	I–IV	Undergoing chemotherapy	IG: 2 (2.22%)
Li and Zhu 2022	China	To explore the effects of conjugal self‐disclosure therapy on cancer recurrence fear and marital quality in young patients with breast cancer	IG: 51 CG: 51	Spouses	/	IG: 33.98 ± 10.13; CG: 34.76 ± 9.80	/	Undergoing chemotherapy	0
Chen and Qin 2023	China	To analyse the effect of follow‐up care based on structured family therapy on FCR and spousal sexual relations of postoperative patients with breast cancer	IG: 12 CG: 12	Spouses	/	/	/	Undergoing surgery, chemotherapy, or radiotherapy	CG: 1 (4.17%)

Abbreviations: CG, control group; IG, intervention group.

### 3.3 Characteristics of the Interventions

The key characteristics of the interventions are summarised and presented in Table 3.

**TABLE 3:** Characteristics of the interventions in the included studies.

Authors	Intervention group										Control group
	Theory	Intervention component			Delivery mode	Teaching and learning modalities	Average time per session	Frequency	Duration	Interveners	
		Psychological support	Disclosure	Education/counselling							
Northouse et al. 2005	Family stress theory	√	√(Some disclosure)	√	Face‐to‐face and phone calls	Home visit, phone calls	1.5 h per visit, 30 min per call	Three home visits, monthly; two follow‐up phone calls	Six months (330 min)	Nurses	Usual care
Huang 2018	/	√	√(Some disclosure)	√	Face‐to‐face and WeChat	Lecture, video, WeChat	/	Five sessions, every three weeks	15 weeks	Nurses	Usual care
Zhang et al. 2019	/		√(Mainly disclosure)		Face‐to‐face and telephone/WeChat	Interview outline	Verbal: 20–30 min, written: 20–30 min	Four sessions	12 weeks (160–240 min)	Nurses	Usual care
Tan 2020	/	√		√	Face‐to‐face	Booklet	/	Every day	Three days (220 min)	Nurses	Usual care
Chen et al. 2022	Positive psychology theory proposed by Seligman		√(Mainly disclosure)		Face‐to‐face	Interview topic	30–60 min	Five sessions, biweekly	Ten weeks (150–300 min)	Trained nurses	Usual care
Li and Zhu 2022	/	√	√	√	Face‐to‐face	Interview outline	Verbal: 25 min; written: 15–25 min	Four sessions	12 weeks (160–200 min)	Nurses	Usual care
Chen and Qin 2023	/		√	√	Face‐to‐face or video software	Home visit, interview outline	1.5 h	Six sessions, weekly	Six weeks (540 min)	Trained nurses	Usual care

#### 3.3.1 Contents of the Interventions

Although the contents of the interventions varied across the seven included studies, they were mainly a mixture of three components with different intensities. These components included disclosure, psychological support and education/counselling.

Disclosure is the expression of feelings, worries, concerns or experiences after being diagnosed with breast cancer. Self‐disclosure is communication by which one person reveals information about themselves to another. This information can be descriptive or evaluative and can include thoughts, feelings, aspirations, goals, failures, successes, fears and dreams, as well as one's likes, dislikes and favourites (Ignatius and Kokkonen 2007). The intervention component that included elements that enabled participants to express their feelings or thoughts was classified as ‘disclosure’. Psychological support was defined as the promotion of the maintenance of an optimistic attitude, whereas education/counselling focused on coping strategies, symptom management and disease‐ or treatment‐related knowledge. Disclosure was the most common component given that six studies, except that by Tan (2020), whose intervention included psychological support and education/counselling, had a disclosure component in their interventions.

Two studies had interventions that mainly involved disclosure between dyads. These studies asked the participants to disclose their feelings and concerns, with the disclosure time ranging from 160 min to 300 min (Zhang et al. 2019; Chen et al. 2022). In addition to disclosure, the intervention in Zhang's et al. (2019) study included a Q&A session to allow participants to ask questions. Two studies employed education/counselling based on disclosure and provided education/counselling specific to their study populations (Chen and Qin 2023; Li and Zhu 2022). Specifically, Li and Zhu's (2022) study integrated psychological support and education on self‐care after treatment and knowledge on disease progression into the intervention for women with breast cancer undergoing chemotherapy. Meanwhile, Chen and Qin's (2023) work integrated sex functioning/relationship and counselling for couples with poor relationships into its intervention.

Compared with interventions that were mainly based on disclosure in four studies, those in the studies by Huang (2018) and Northouse et al. (2005) comprised a large proportion of psychological support and a small proportion of disclosure (i.e., some disclosure). The intervention in the study by Northouse et al. (2005) mainly included education and psychological support and had five core content areas: Family involvement, optimistic attitude, coping effectiveness, uncertainty reduction and symptom management. Huang's (2018) study mainly provided education on disease‐ and treatment‐related knowledge and coping strategies to patients and caregivers, with a small proportion of disclosure in terms of encouraging women to disclose.

In terms of the mode of disclosure, two studies used verbal and written disclosure (Li and Zhu 2022; Zhang et al. 2019), whereas the other four studies focused on verbal disclosure only (Chen et al. 2022; Chen and Qin 2023; Huang 2018; Northouse et al. 2005). All the participants in the control group were treated with usual care for cancer management.

Only two studies had interventions that were formulated on the basis of theoretical frameworks, including family stress theory and positive psychology theory (Chen et al. 2022; Northouse et al. 2005). The detailed contents of the interventions are provided in Table 3.

#### 3.3.2 Dosage of the Interventions

The dosage of an intervention involves amount, frequency and duration. The amount is the number of sessions, frequency is the number of times the intervention is delivered in a specified time period, and duration is the total duration time of the intervention (Reed et al. 2007). Therefore, the dosage of the included interventions was summarised on the basis of these three aspects. Two studies did not provide detailed intervention dosages in terms of each session (Huang 2018; Tan 2020). The interventions in the other five studies comprised weekly to monthly sessions. Each session lasted for 30 min to 1.5 h. The total durations of the interventions reported in the included studies ranged from 3 days to 6 months, with estimated intervention durations ranging from 150 min to 540 min in total (Table 3).

#### 3.3.3 Delivery Modes, Formats and Interventionists

The interventions were administered through different modes, including face‐to‐face, WeChat, phone calls and video software. All six of the studies in China were couple‐based, whereas that in the USA was family‐based (Northouse et al. 2005). Four studies solely provided face‐to‐face sessions (Chen et al. 2022; Huang 2018; Li and Zhu 2022; Tan 2020), whereas the other three employed face‐to‐face sessions combined with phone calls, WeChat, or video software (Chen and Qin 2023; Northouse et al. 2005; Zhang et al. 2019). For the venues, two studies conducted interventions via home visits (Chen and Qin 2023; Northouse et al. 2005), and the other five studies performed interventions in hospitals. Teaching and learning modalities included written materials, videos and interview outlines. Each study adopted a different combination of these modalities, as shown in Table 3. All the interventions were delivered by nurses. Two studies (Chen et al. 2022; Chen and Qin 2023) reported that the nurses were trained specially for the study.

### 3.4 Risk of Bias of the Studies

Seven RCTs were assessed by using ROB‐2 (Higgins et al. 2019). Table 4 provides a summary of the risk of bias in the included studies. The table shows the results of judgements of the individual risk of bias for each included study and presents the worst‐case scenario for each study in reference to the outcome for which the risk of bias was high. The overall risk of bias was high in one study (Northouse et al. 2005) and was of some concern in six studies (Chen et al. 2022; Chen and Qin 2023; Huang 2018; Li and Zhu 2022; Tan 2020; Zhang et al. 2019).

**TABLE 4:** Risk of bias of the RCTs.

Author	Randomisation	Deviations from the intended interventions	Missing outcome data	Measurement of the outcome	Selection of the reported result	Overall
Northouse et al. 2005	Some concerns	Some concerns	High	Some concerns	Some concerns	High
Huang 2018	Some concerns	Some concerns	Low	Some concerns	Some concerns	Some concerns
Zhang et al. 2019	Some concerns	Some concerns	Low	Some concerns	Some concerns	Some concerns
Tan 2020	Some concerns	Some concerns	Low	Some concerns	Some concerns	Some concerns
Chen et al. 2022	Some concerns	Some concerns	Low	Some concerns	Some concerns	Some concerns
Li and Zhu 2022	Some concerns	Some concerns	Low	Some concerns	Some concerns	Some concerns
Chen and Qin 2023	Some concerns	Some concerns	Low	Some concerns	Some concerns	Some concerns

For the domain of randomisation, all studies did not mention the allocation concealment. Two studies reported allocating participants in accordance with a random number table (Huang 2018; Tan 2020), three studies reported allocating participants in accordance with their admission sequence (Chen and Qin 2023; Zhang et al. 2019) or ward (Chen et al. 2022), and one study did not describe their allocation mechanism (Northouse et al. 2005).

In terms of deviations from the intended intervention domain, biases arise when deviations exist from the intended interventions. All studies were assessed as having some concerns because of the absence of a protocol for assessing whether the administration of additional interventions was inconsistent with the trial protocol, failure to implement the protocol interventions as intended, or nonadherence by trial participants to their assigned intervention.

For the domain of bias in missing outcome data, one study showed a high risk of bias because it had imbalances in missing data across groups, and no appropriate methods were applied to address the potential bias (Northouse et al. 2005). In accordance with the Cochrane handbook, a proportion of less than 5% missing outcome data is considered small (Higgins et al. 2019). Other studies were rated as having some concerns.

With respect to the domain of bias in outcome measurement, all the included studies were rated as having some concerns because the outcome assessment was potentially influenced by the knowledge of the intervention received. Moreover, although the study outcomes were measured by a self‐reported questionnaire, all the studies did not report whether the outcome assessor was blinded to group allocation or not (Chen et al. 2022; Chen and Qin 2023; Huang 2018; Li and Zhu 2022; Northouse et al. 2005; Tan 2020; Zhang et al. 2019). One study used an electronic questionnaire (Chen and Qin 2023), and three studies collected data face‐to‐face via questionnaires without reporting blinding (Chen et al. 2022; Li and Zhu 2022; Zhang et al. 2019).

One study collected data via an e‐questionnaire and a face‐to‐face questionnaire (Huang 2018). One study did not describe how its data were collected (Tan 2020).

Regarding the domain of the selection of the reported result, all the included studies were considered to have some concerns due to the lack of preregistered trial protocols (Chen et al. 2022; Chen and Qin 2023; Huang 2018; Li and Zhu 2022; Northouse et al. 2005; Tan 2020; Zhang et al. 2019).

### 3.5 Effects of Family Involvement Interventions

#### 3.5.1 Study Outcome Assessment

Three different FCR scales, namely, Mishel's Uncertainty in Illness Scale (MUIS), Fear of Progression Questionnaire‐Short (FoP‐Q‐SF) and Fear of Cancer Recurrence Inventory Chinese Version (FCRI‐CV), were used to assess patients' FCR level. One scale, namely, MUIS, was used to assess caregivers' FCR level. The above three scales have been validated. Table 5 summarises the outcome measurement tools and key findings across all the seven studies. All the included studies measured outcomes immediately postintervention without a follow‐up period.

**TABLE 5:** FCR measurement tools and outcomes.

Author, year	Women with breast cancer			Caregivers'		
	FCR measurement	Assessment point	Key finding	FCR measurement	Duration	Key findings
Northouse et al. 2005	MUIS	Postintervention	FCR↓(*p* > 0.05)	MUIS	Postintervention	FCR↓ (*p* > 0.05)
Huang 2018	FoP‐Q‐SF	Postintervention	FCR↓ (*p* < 0.001)	/	/	/
Zhang et al. 2019	FoP‐Q‐SF	Postintervention	FCR↓ (*p* < 0.001)	/	/	/
Tan 2020	FoP‐Q‐SF	Postintervention	FCR↓ (*P* < 0.05)	/	/	/
Chen et al. 2022	FoP‐Q‐SF	Postintervention	FCR↓ (*p* = 0.001)	/	/	/
Li and Zhu 2022	FoP‐Q‐SF	Postintervention	FCR↓ (*P* < 0.001)	/	/	/
Chen and Qin 2023	FCRI‐CV	Postintervention	FCR↓ (*P* < 0.05)	/	/	/

Abbreviations: FCRI‐CV, Fear of Cancer Recurrence Inventory Chinese Version; FoP‐Q‐SF, Fear of Progression Questionnaire‐Short; MUIS, Mishel's Uncertainty in Illness Scale.

#### 3.5.2 Women With Breast Cancer

##### 3.5.2.1 Post‐intervention

**FIGURE 2:**
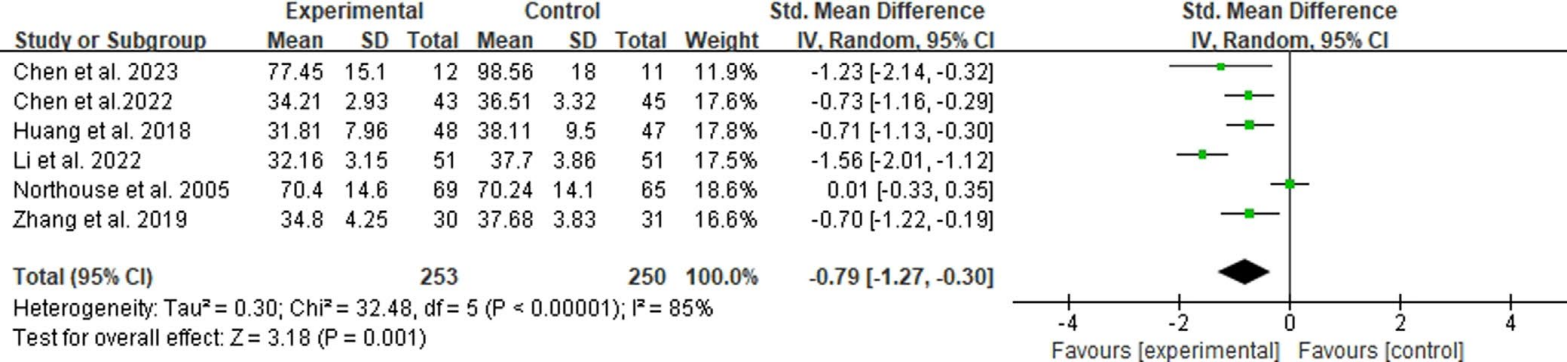
Effects of family involvement interventions on FCR in women with breast cancer at postintervention. [Colour figure can be viewed at wileyonlinelibrary.com]

All the seven studies reported alleviation in FCR in women with breast cancer. In most studies, except for the work of Northouse et al. (2005), the reductions were statistically significantly greater in the intervention group than in the control group. One study was excluded from the meta‐analysis because the mean and SD of FCR were not reported (Tan 2020). Therefore, six studies involving 503 women with breast cancer that evaluated the effectiveness of family involvement interventions on FCR levels at postintervention were included in the meta‐analysis (Chen et al. 2022; Chen and Qin 2023; Huang 2018; Li and Zhu 2022; Northouse et al. 2005; Zhang et al. 2019). Substantial heterogeneity was observed (*p* < 0.0001; *I* ^2^ = 78%). Hence, a random‐effects model was used. Figure 2 shows the meta‐analysis of the intervention effect on FCR in women with breast cancer. The pooled results demonstrated a significantly larger reduction in the FCR of women with breast cancer immediately after receiving the family involvement interventions than in the control group (SMD = −0.79, 95% CI: [−1.27, −0.30], *p* = 0.001).

##### 3.5.2.2 Subgroup Analysis by Intervention Component

**FIGURE 3:**
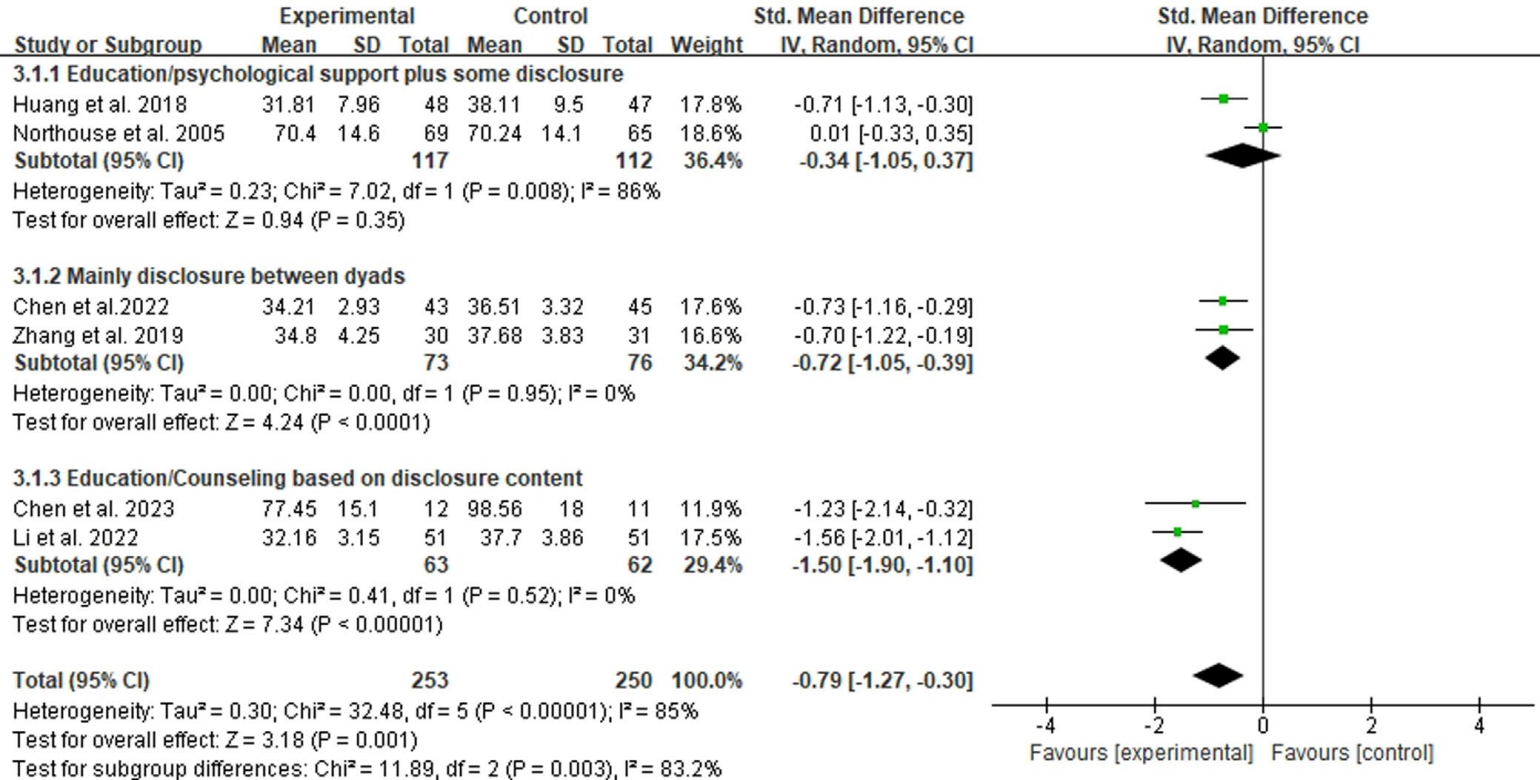
Effects of family involvement intervention on FCR in women with breast cancer at postintervention in subgroups. [Colour figure can be viewed at wileyonlinelibrary.com]

All the six studies included in the meta‐analysis included a disclosure component in their interventions. Three subgroups were created for analysis in accordance with disclosure intensity and additional components. Two studies involving 229 women with breast cancer and using education/psychological support plus some disclosure (Huang 2018; Northouse et al. 2005) showed substantial heterogeneity (*p* = 0.008; *I* ^2^ = 86%). Two studies (Chen et al. 2022; Zhang et al. 2019) employing solely disclosure between dyads involving 149 women with breast cancer and two studies (Chen and Qin 2023; Li and Zhu 2022) using education/counselling based on disclosure content involving 125 women with breast cancer showed no heterogeneity (*p* > 0.05; *I* ^2^ = 0). A random‐effects model was employed. Figure 3 shows the meta‐analysis of the three subgroups for FCR in women with breast cancer. The pooled results demonstrated that compared with usual care, education/psychological support plus some disclosure was ineffective (SMD = −0.34, 95% CI: [−1.05, 0.37], *p* = 0.35) and disclosure mainly had a moderate‐to‐large effect size (SMD: −0.72, 95% CI: [−1.05, −0.39], *p* < 0.00001), whereas disclosure based on education or counselling targeting the specific needs of the participants had an extremely large effect size (SMD: −1.50, 95% CI: [−1.90, −1.10], *p* < 0.00001). For the two RCTs in the third category of disclosure based on education or counselling, the target participants in Li and Zhu's (2022) study were patients with breast cancer undergoing chemotherapy, and education on chemotherapy‐related information was provided. Meanwhile, the dyads in Chen and Qin's (2023) study had poor relationships due to sexual functioning, and sexual counselling was provided by a psychologist. The subgroup analysis showed significant differences across subgroups (*p* = 0.003) (Figure 3).

##### 3.5.2.3 Sensitivity Analysis

In the sensitivity analysis, which excluded one study each time for the comparison of family involvement interventions and usual care at postintervention, the pooled intervention effect fluctuated between −0.96 (95% CI: [−1.33, −0.59]) and − 0.6 (95% CI: [−0.99, −0.20]). The pooled effect sizes remained significant when one of the trials was excluded, indicating that the overall significant result of the comparison was robust and not influenced by any study.

#### 3.5.3 Caregivers

Only one study (Northouse et al. 2005) evaluated the effectiveness of the family involvement intervention in FCR in caregivers at post‐intervention (i.e., at 6 months). Although the intervention group showed a greater reduction in the mean scores in FCR in caregivers at postintervention (reduced by 5.39 points) than in the control group (reduced by 2.6 points), the between‐group difference was statistically insignificant (*p* > 0.05).

## 4 Discussion

To the best of the authors' knowledge, this systematic review is the first to examine the effectiveness of family involvement interventions on FCR alleviation in women with breast cancer and their caregivers. FCR is common and severe in women with breast cancer and their caregivers, and their FCR levels can be affected by each other (Janz et al. 2016; Soriano et al. 2021). Seven studies were included in this review. Although caregivers were involved in the interventions in all seven studies, only one high‐risk study (Northouse et al. 2005) reported FCR in caregivers, and the effectiveness of the intervention (education/psychological support plus some disclosure) in caregivers was insignificant, likely due to the exclusion of data from dyads with patients having stage 1 and 2 cancer from the analysis. Additionally, nearly 20% of the participants died prior to the completion of the study. Although a proportion of over 20% missing outcomes is traditionally regarded as large (Higgins et al. 2019), a 20% attrition rate is still acceptable given that the attrition rate in metastatic breast cancer has been reported to be between 9% and 53% (Nuzzolese and Montemurro 2020). The intervention in the study of Northouse et al. (2005) may have had little time for disclosure for caregivers to share fears and concerns, given that it covered numerous aspects (e.g., coping, symptom management, education and psychological support) and was supplemented with a 17‐page protocol manual, suggesting that the time for disclosure could be increased to improve the results for FCR in caregivers (Northouse et al. 2005). Nevertheless, FCR reduction in the caregivers of women with breast cancer is under‐researched.

The results of the meta‐analysis revealed that family involvement interventions had significant short‐term effects on FCR in women at postintervention, with an overall moderate effect size. The results of the subgroup analysis showed that disclosure involving patients with breast cancer and their family members should be a major interventional component in reducing FCR. Disclosure can reduce barriers to expressing emotions for those with constraints on disclosure (Ignatius and Kokkonen 2007). This effect could be a main reason for the observed favourable result of family involvement interventions. The literature has consistently reported that people experience barriers in expressing their emotions, especially when facing social constraints (Soriano et al. 2021, 2018). Social constraints are negative social responses that can lead to the avoidance of thinking or talking about cancer. They may inhibit cognitive processing and exacerbate emotional distress (Soriano et al. 2021). Social constraints on disclosure are a type of social constraint that is frequently reported in studies (Soriano et al. 2021, 2018). Studies have shown that women and their family members have social constraints on disclosure and find expressing their true emotions and feelings difficult (Soriano et al. 2021; Wang et al. 2020). They may hide worries, deny concerns and yield to their family members in an effort to avoid disagreement and reduce their upset and burden (Manne et al. 2007). When a patient or caregiver perceives social constraints on the disclosure of cancer‐related concerns, they may not express their thoughts and their cognitive processing is incomplete; they then experience increased FCR (Soriano et al. 2021). High heterogeneity was observed in the included studies. The small fluctuation in effect sizes and consistency of the statistically significant results across all sensitivity analyses suggest that the observed heterogeneity is likely due to factors other than the influence of individual studies. These factors may include intervention design (e.g., intensity, duration, or delivery format), participant characteristics (e.g., age, cancer stage, or treatment), or outcome measurement tools. They could be explored in great depth in future studies to understand their effect on heterogeneity.

Six studies used disclosure. Five of these studies were from China and had promising results likely because the Chinese cultural value of emotional suppression to avoid disturbing the harmonious equilibrium of interpersonal relationships imposes barriers on the participants' ability to express emotions and leads to their refusal to communicate with others (Wang et al. 2020). Furthermore, in China, breast cancer is considered as incurable and as a death sentence due its cultural and historical stigma in society (Wang et al. 2020). Consequently, women and their caregivers are compelled to keep their cancer diagnosis to themselves to avoid stigma and do not discuss the disease with friends or family because doing so would be perceived as vanity or met with disapproval and lack of sympathy (Wang et al. 2020; Zhu et al. 2012). Disclosure between women with breast cancer and their caregivers solved the communication problem and provided a supportive environment. Talking with others may facilitate the cognitive processing of breast cancer experiences. Benefits should be accrued if talking is met with supportive, receptive, or noncritical social responses but not if it is met with unsupportive, unreceptive or critical social responses. Supportive, or empathic, social networks help people maintain or re‐establish a positive self‐concept because such responses validate people's experiences and affirm that they are loved and esteemed. Additional rigorous RCTs are needed to support this assertion because all the included studies in the meta‐analyses, including one with high risk (Chen et al. 2022; Chen and Qin 2023; Northouse et al. 2005; Zhang et al. 2019) and six studies with some concerns (Huang 2018; Li and Zhu 2022; Tan 2020), had methodological weaknesses, in particular, unclear random sequence generation, allocation concealment, masking of outcome assessors and inappropriate analysis of missing outcome data.

In addition, the present meta‐analysis found that disclosure combined with education or counselling targeting the specific needs of participants produced an extremely large pooled effect size on reducing FCR in women with breast cancer. This result suggests that disclosure supplemented with education or support that is related to the cancer experience could further enhance the effect of the intervention. Some healthy lifestyle components, such as physical exercise and nutrition, could be considered to develop interventions that can be applicable to most patients with breast cancer and their caregivers. Supplementary education on healthy lifestyles may further enhance the intervention effect. First, education on healthy lifestyles for cancer management can equip dyads with knowledge (Zhou et al. 2024) that they can easily understand to increase their topics during disclosure. Second, increased physical exercise and healthy eating can reduce illness representation and cancer recurrence risk in patients with breast cancer (Chlebowski et al. 2006; Jochems et al. 2018). This effect, in turn, can reduce FCR in patients and then in caregivers.

Notably, the interventions in the included studies were delivered via mainly a face‐to‐face format or a combination of face‐to‐face format with telephone calls, WeChat or video software. In consideration of the wide accessibility of smartphones and the Internet, technology‐based delivery could enable the administration of interventions to caregivers with increased time and location flexibility because caregivers of patients with cancer usually have financial concerns and work commitments that make attending face‐to‐face interventions difficult. A previous systematic review showed that an Internet‐based intervention programme for caregivers of older adults could improve caregivers' mental health (Sherifali et al. 2018). Therefore, whether a technology‐based intervention for dyads in the context of patients with cancer and their caregivers is feasible and effective is worth exploring. Further studies are needed to explore interventions with the most appropriate mode and intensity for their sustainability to achieve long‐term effects. In addition, no study in the present review reported the long‐term effects of family involvement interventions for FCR alleviation in women with breast cancer and their caregivers.

### 4.1 Limitations

This review has some limitations. Firstly, the majority of the included studies had methodological weaknesses, such as unclear sequence randomisation, allocation concealment, blinding of the data collectors and inappropriate analysis of missing outcome data. Such weaknesses might have imposed bias on the pooled results from the meta‐analysis. Secondly, most of the studies were conducted in China and hence limited the generalisability of the review findings. Thirdly, one study was excluded from the meta‐analysis because the means and SDs of FCR were not reported. We attempted to access data from the authors; however, their email addresses were not provided. The missing data may distort the true effects. Lastly, publication bias was not evaluated given that fewer than 10 studies were included.

### 4.2 Implications for Practice

This review found that family involvement interventions have considerable short‐term effects on FCR in women with breast cancer. Therefore, in practice, family involvement interventions can be considered for delivery to patient–caregiver dyads to improve FCR in women with breast cancer. In this review, all the interventionists were nurses. This situation indicates that nurses can play a promising role in the administration of FCR interventions to women with breast cancer. Given that nurses have close contacts with patients with cancer and their caregivers, the interventions that they deliver are highly likely to be integrated into clinical practice. Nurses should enhance their skills in educating and establishing therapeutic relationships with patients with breast cancer and their caregivers to help them implement interventions effectively and facilitate the delivery of nurse‐led interventions in clinical practice. In addition, caregivers undertake caring responsibilities at home over a long duration. How health care providers can optimally implement interventions requires further investigation. In this review, most of the interventions were delivered face‐to‐face when the women underwent treatment. Not many studies were delivered through home visits, WeChat, telephone calls, and video software, thus posing a limitation. Therefore, additional acceptable and flexible delivery modes, such as a combination of face‐to‐face and technology‐based formats, need to be considered in clinical practice.

### 4.3 Implications for Research

In consideration of the methodological weaknesses of some studies, future RCTs need rigorous designs, such as clear random sequence generation, allocation concealment and blinding of the data collector, and appropriate analysis of missing outcome data. In this review, studies were conducted in China and the USA; additional research is needed in other countries. All the studies lacked follow‐up. Hence, further research is needed to explore the long‐term effects of the interventions. Only a few interventional studies targeted patients and caregivers. Although caregivers experienced a high level of FCR, only one study measured FCR in caregivers. Given that FCR in women with breast cancer and their caregivers are influenced by each other, many studies have suggested reducing FCR in women with breast cancer and their caregivers by using a dyadic perspective (Janz et al. 2016; Soriano et al. 2021) or addressing FCR in caregivers (Boehmer et al. 2016). Given that physical activity and a healthy diet can reduce cancer recurrence, future studies may explore interventions based on disclosure supplemented with education on behavioural components (e.g., physical activity and a healthy diet) to reduce FCR from a family perspective. These components can reduce FCR by helping patients and caregivers to perceive a low risk of cancer recurrence in patients and generate topics for disclosure.

## 5 Conclusions

The review findings indicate that family involvement interventions, especially those using disclosure combined with education or counselling targeting the specific needs of the participants, may have remarkable short‐term effects on FCR alleviation in women. The results of family involvement intervention for caregivers showed no significance in one study, and only a few interventional studies targeted patients and caregivers. The main contents of effective family involvement interventions included contents related to disclosure, education/psychological support plus some disclosure and education/counselling based on disclosure content. This study showed that in contrast to interventions involving education/psychological support plus some disclosure, those encompassing disclosure combined with education or counselling and those mainly including disclosure produced promising effects to reduce FCR in women. Intervention varied in content, delivery mode, frequency and format. Further high‐quality RCTs on family involvement for women with breast cancer and/or their caregivers with follow‐ups are encouraged to generate adequate evidence.

## Conflicts of Interest

The authors declare no conflicts of interest.

## Supporting information


Appendix S1.

## Data Availability

Data sharing is not applicable to this work because no new data were created or analysed in this study.
